# Oxolipidomics profile in major depressive disorder: Comparing remitters and non-remitters to repetitive transcranial magnetic stimulation treatment

**DOI:** 10.1371/journal.pone.0246592

**Published:** 2021-02-11

**Authors:** Hannah Stirton, Benjamin P. Meek, Andrea L. Edel, Zahra Solati, Arun Surendran, Harold Aukema, Mandana Modirrousta, Amir Ravandi

**Affiliations:** 1 Cardiovascular Lipidomics Laboratory, St. Boniface Hospital Albrechtsen Research Centre, Winnipeg, Manitoba, Canada; 2 Max Rady College of Medicine, University of Manitoba, Winnipeg, Manitoba, Canada; 3 Dept. of Psychiatry, University of Manitoba, Winnipeg, Manitoba, Canada; National Institutes of Health, UNITED STATES

## Abstract

**Background:**

Repetitive Transcranial Magnetic Stimulation [rTMS] is increasingly being used to treat Major Depressive Disorder [MDD]. Given that not all patients respond to rTMS, it would be clinically useful to have reliable biomarkers that predict treatment response. Oxidized phosphatidylcholine [OxPC] and some oxylipins are important plasma biomarkers of oxidative stress and inflammation. Not only is depression associated with oxidative stress, but rTMS has been shown to have anti-oxidative effects.

**Objectives:**

To investigate whether plasma oxolipidomics profiles could predict treatment response in patients with treatment resistant MDD.

**Methods:**

Fourty-eight patients undergoing rTMS treatment for MDD were recruited along with nine healthy control subjects. Plasma OxPCs and oxylipins were extracted and analyzed through high performance liquid chromatography coupled with mass spectrometry. Patients with a Hamilton Depression Rating Scale score [Ham-D] ≤7 post-treatment were defined as having entered remission.

**Results:**

Fifty-seven OxPC and 32 oxylipin species were identified in our subjects. MDD patients who entered remission following rTMS had significantly higher pre-rTMS levels of total and fragmented OxPCs compared to non-remitters and controls [one-way ANOVA, p<0.05]. However, no significant changes in OxPC levels were found as a result of rTMS, regardless of treatment response [p>0.05]. No differences in plasma oxylipins were found between remitters and non-remitters at baseline.

**Conclusion:**

Certain categories of OxPCs may be useful predictive biomarkers for response to rTMS treatment in MDD. Given that elevated oxidized lipids may indicate higher levels of oxidative stress and inflammation in the brain, patients with this phenotype of depression may be more receptive to rTMS treatment.

## Introduction

Major Depressive Disorder [MDD] is a debilitating psychiatric condition that has a lifetime prevalence of 20.6% [1]. Over 300 million people are affected by depression and it is the leading cause of disability worldwide [2]. MDD increases the risk of developing diabetes mellitus, heart disease and stroke, further contributing to the burden of disease [3].

The role of oxidative stress as a potential cause for MDD is beginning to gain more traction in the research world [4]. Oxidative stress occurs when there is an imbalance between oxidant and antioxidant processes in the body [5]. Reactive oxygen species [ROS] are one major source of oxidative stress, produced as by-products from normal cellular respiration [6]. Excessive ROS can react with many biological molecules, including lipids, proteins and DNA, causing lipid peroxidation, protein cleavage and DNA mutation [6]. Brain cells are highly vulnerable to oxidative stress because the brain is composed of at least 40% lipids, consumes 20% of total body oxygen, and has low anti-oxidant defences [4, 7–9]. Several studies have proposed that increased oxidative stress may be a contributing factor in the pathogenesis of MDD [10–12]. Patients with MDD have higher levels of certain oxidative stress biomarkers, including F2-isoprostanes and 8-OH 2-deoxyguanosine [8-OHdG] [13].

**Fig 1:**
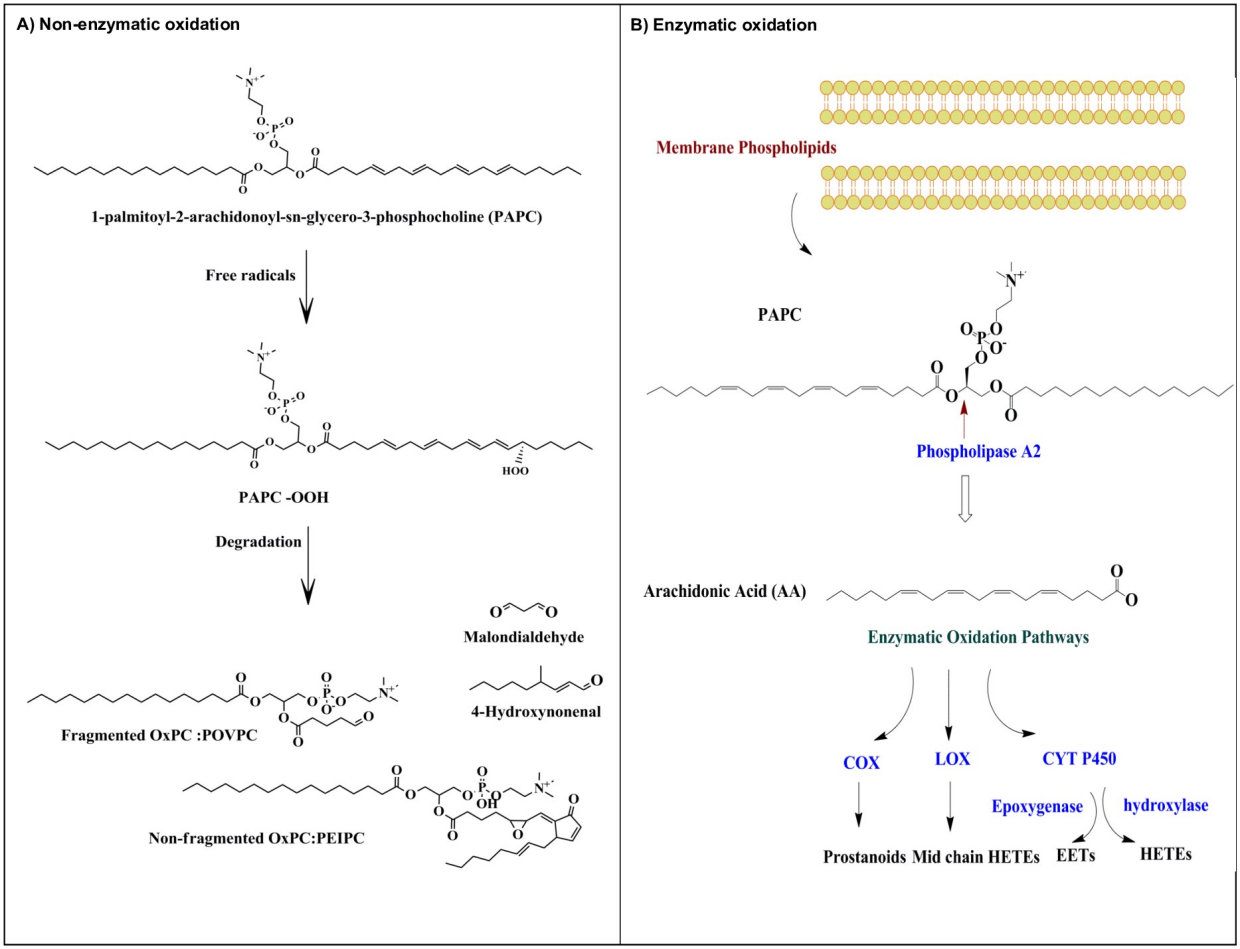
**A]** Non-enzymatic oxidation of membrane phospholipids. Free radicals may attack membrane phospholipids such as PAPC, leading to the production of bioactive lipid molecules. Abbreviations: PAPC-OOH, PAPC hydroproxide; OxPC, oxidized phosphatidylcholine; PEIPC, 1-palmitoyl-2-[5,6-epoxyisoprostane E2]-sn-glycero-3-phosphocoline. **B]** Enzymatic oxidation of membrane phospholipids. Fatty acids are released from the membrane phospholipid by the phospholipase A2 enzyme and may undergo oxidation through three oxidation pathways including COX, LOX, and CYT P450. Abbreviations: COX, cyclooxygenase; LOX, lipoxygenase; CTYP450, cytochrome P450; HETE; hydroxyeicosatetraenoic acids; EET, epoxyeicosatrienoic acids.

Oxidized phosphatidylcholines [OxPCs] are one of the most abundant and well known classes of the oxidized phospholipids [OxPLs] [14]. When phosphatidylcholine [an important building block of cell membranes] is oxidized, a heterogenous pool of end-products are formed, including fragmented and non-fragmented OxPCs [Fig 1a] [15]. Thus, OxPCs represent an important set of oxidative stress biomarkers and can cause organ damage at high levels. While some research has been done on the relationship between depression and oxidative stress, to our knowledge, no study has yet examined the OxPC profile in patients with MDD [13].

Inflammation is another biological process that has been linked to the development of depression [11]. Oxylipins are a class of highly bioactive molecules that are known to play a role in inflammation [16]. Oxylipins are oxidation products formed from polyunsaturated fatty acids [PUFAs] by 3 main enzymes, including cyclooxygenase [COX], lipoxygenase [LOX] and cytochrome P450 [CYP] [Fig 1b], although some are derived non-enzymatically. While some oxylipins are thought to be anti-inflammatory, others show pro-inflammatory characteristics and are therefore thought to be implicated in MDD [11]. This suggests oxylipin levels and profiles may be altered in certain patients with depression.

Repetitive Transcranial Magnetic Stimulation [rTMS] is a relatively new treatment option that was approved by the FDA in 2008 for the treatment of MDD [17]. Response rates to rTMS are reported to be between 45 and 60%, with remission rates ranging from 30 to 40% [18]. Why certain patients respond to rTMS, while others do not, remains largely unknown. Given this variable response to rTMS, as well as its expensive and time-consuming nature, it would be clinically useful to have reliable biomarkers that predict treatment response. An ideal biomarker would be one that links the possible treatment mechanism of rTMS to an underlying pathophysiology of depression. Interestingly, rTMS has been shown to reduce oxidative stress and inflammation in the brain [19–22].

Lipidomics represents a promising approach to identify new diagnostic biomarkers in the context of MDD. As lipids are able to cross the blood-brain barrier, products of oxidative stress and inflammation in the brain can be measured through lipidomics analysis of blood plasma [23]. We performed exploratory analyses to achieve three objectives. First, to determine if pre-rTMS oxolipidomics profile [OxPCs and oxylipins] differed between MDD patients who entered remission after rTMS and those who did not. Our second objective was to see if there were differences in OxPC profiles before and after a course of rTMS, and if these differences corresponded to clinical changes in depressive symptoms. Our third objective was to determine if OxPC profiles differed between subjects with MDD and non-depressed controls.

## Methods and materials

### Patient recruitment and sample size

Fourty-eight patients undergoing rTMS treatment for MDD were recruited along with nine control subjects. Recruitment took place from June 2015 to July 2018. Control participants, aged 18–80, were recruited through advertising flyers posted at research institutes in Winnipeg. Healthy controls were screened by research staff prior to participation for any current or prior diagnosis of depression, as well as the list of exclusion criteria described for patients. Informed consent was obtained prior to initiation of study procedures. Non-depressed controls were screened and excluded if they had any active psychiatric diseases. Patients were recruited from the pool of rTMS referrals to the Neuromodulation and Neuropsychiatry Unit at St. Boniface Hospital. Eligible candidates were identified by the participating psychiatrist. Eligible subjects were patients with a major depressive episode between 18–80 years old who were not actively receiving psychotherapy or electroconvulsive therapy [ECT]. Subjects were excluded if they had a history of a psychotic episode, neurological illness, traumatic brain injury, active alcohol or substance abuse, seizure disorder or were pregnant. The total sample size was 57 [including controls] for the OxPC analysis, while the sample size for oxylipin analysis was 46. The study was approved by the ethics committee of both the University of Manitoba [HS18975] and the St. Boniface Hospital [RRC/2015/1449] research ethics boards. Written informed consent was obtained from patients prior to their inclusion in the study.

### rTMS treatment

Patients referred to this clinic were screened by a psychiatric nurse for the standard exclusion criteria for rTMS therapy: metal in the body, a family or personal history of epilepsy, brain tumor, or any other major neurological disorder. Patients were given a full psychiatric interview by the Unit psychiatrists before treatment, which included screening for disease-specific exclusion criteria such as psychotic depression, active suicidal ideations, Persistent Depressive Disorder with no significant remission, and previous failure to ECT and/or rTMS therapy. All eligible candidates met *Diagnostic and Statistical Manual of Mental Disorders*, *Fifth Edition* [*DSM-5*] diagnostic criteria for MDD and did not have active substance use disorder or any past or current primary psychotic disorders [24]. Patients scheduled to receive rTMS in this clinic were required to keep a stable medication regimen [no changes in medication or dosage] for four weeks before the start of rTMS and to maintain this stability for the duration of treatment. The 17-item Hamilton Rating Scale for Depression [Ham-D] was administered to MDD subjects by the treating psychiatrists within 1 week before the first session of rTMS and then again after every 10 sessions of treatment to monitor changes in symptoms [25]. After 20 sessions, patients were discontinued from treatment if they showed no change [or a worsening] of Ham-D score from baseline. rTMS was delivered via Rapid2 magnetic stimulator [Magstim Co., UK]. The majority of patients received either high-frequency or theta-frequency rTMS protocol as part of their treatment, which targets the left dorsolateral pre-frontal cortex [DLPFC] as determined by the high-resolution 3-dimensional [3D] T1-weighted MRI scans using BrainSight navigator [Rogue Research Inc.QC]. Three patients received low-frequency protocol rTMS which targets the right DLPFC. All protocols involved a total of 30 sessions over 15 consecutive working days at 110% of motor-threshold. Each session in high frequency protocol involved 50 short trains of magnetic pulses, each of which consisted of 60 pulses with a frequency of 10 Hz [for a total of 3000 pulses in one session], with 25-second intervals between stimulation trains. Each session in theta protocol involved 30 trains of pulses, each of which consisted of 10 bursts at 5Hz of 3 pulses each at 50Hz [for a total of 900 pulses/session], with 10-second inter-train intervals. Each session in low frequency protocol involved 20 trains of magnetic pulses, each of which consisted of 60 pulses at 1Hz [for a total of 1200 pulses/session] with 30-second inter-train intervals. After rTMS treatment, patients were classified as either remitters or non-remitters based on their post-treatment Ham-D score. Regardless of baseline scores, patients were classified as entering remission if their post-treatment Ham-D score was 7 or less, a number widely accepted within the medical and research community as indicative of MDD remission [26].

### Sample collection

Fasting blood samples [10ml] were drawn from subjects on two occasions: i] within one week prior to rTMS treatment and ii] at the end of rTMS treatment. Blood was drawn in sodium EDTA tubes and plasma was obtained from whole blood via centrifugation for 15 min at 2500 rpm. Plasma samples were capped and stored at −80°C until lipid extraction was performed. For control patients, fasting blood [10 ml] was drawn on one occasion as they did not receive rTMS treatment.

### Blood sample analysis

OxPCs from plasma were extracted by a method using 2:1 [vol/vol] chloroform:methanol [CM] previously described by Folch et al. with modifications [27]. Briefly, 850 μL of CM containing 0.01% butylated hydroxytoluene [BHT] was added to 100μL plasma, along with 100 μL of an internal standard, 9:0 phosphatidylcholine [PC 0.1 μg/ml]. Ninety microliters of PBS was then added to this mixture, which were then centrifuged at 4°C for 5 min at 2500 rpm. The lower lipid phase was extracted, while the remaining aqueous phase was re-reconstituted in 600 μL of ice cold chloroform. This process of centrifugation and lipid extraction was then repeated twice more. The organic phase was evaporated under a nitrogen evaporator and then re-dissolved in 100 μL of Solvent A [water-acetonitrile-formic acid [63:37:0.02; v/v/v]] prior to immediate LC-MS analysis. For the oxylipin extraction, 200 μL of plasma was spiked with 100 μL of oxylipin internal standard and 100 μL of antioxidant cocktail [0.2 mg/ml BHT, 0.2 mg/ml EDTA, 2 mg/ml triphenylphosphine, 2 mg/ml indomethacin in a solution of 2:1:1 methanol:ethanol:H_2_O]. Samples were acidified to pH3 prior to centrifugation at 4°C for 10 min. Oxylipins were extracted using Strata X SPE 60mg/3ml columns [Phenomenex, Torrance, CA]. Columns were washed with 3.5 ml of 100% methanol followed by 3.5 ml of pH3 water. The sample was then added to the column and the vials were washed with 1 ml of 10% methanol in pH3 water, re-centrifuged for 5 min, and re-applied to the columns. The columns were washed with 2 ml of pH3 water and dried with 1 ml hexane. The oxylipins were eluted with 1 ml methanol and the eluent was dried under nitrogen gas and re-dissolved in 100 μL of Solvent A [water-acetonitrile-acetic acid [70:30:0.2; v/v/v]] for immediate LC-MS analysis.

### High performance liquid chromatography and mass spectrometry

**Fig 2:**
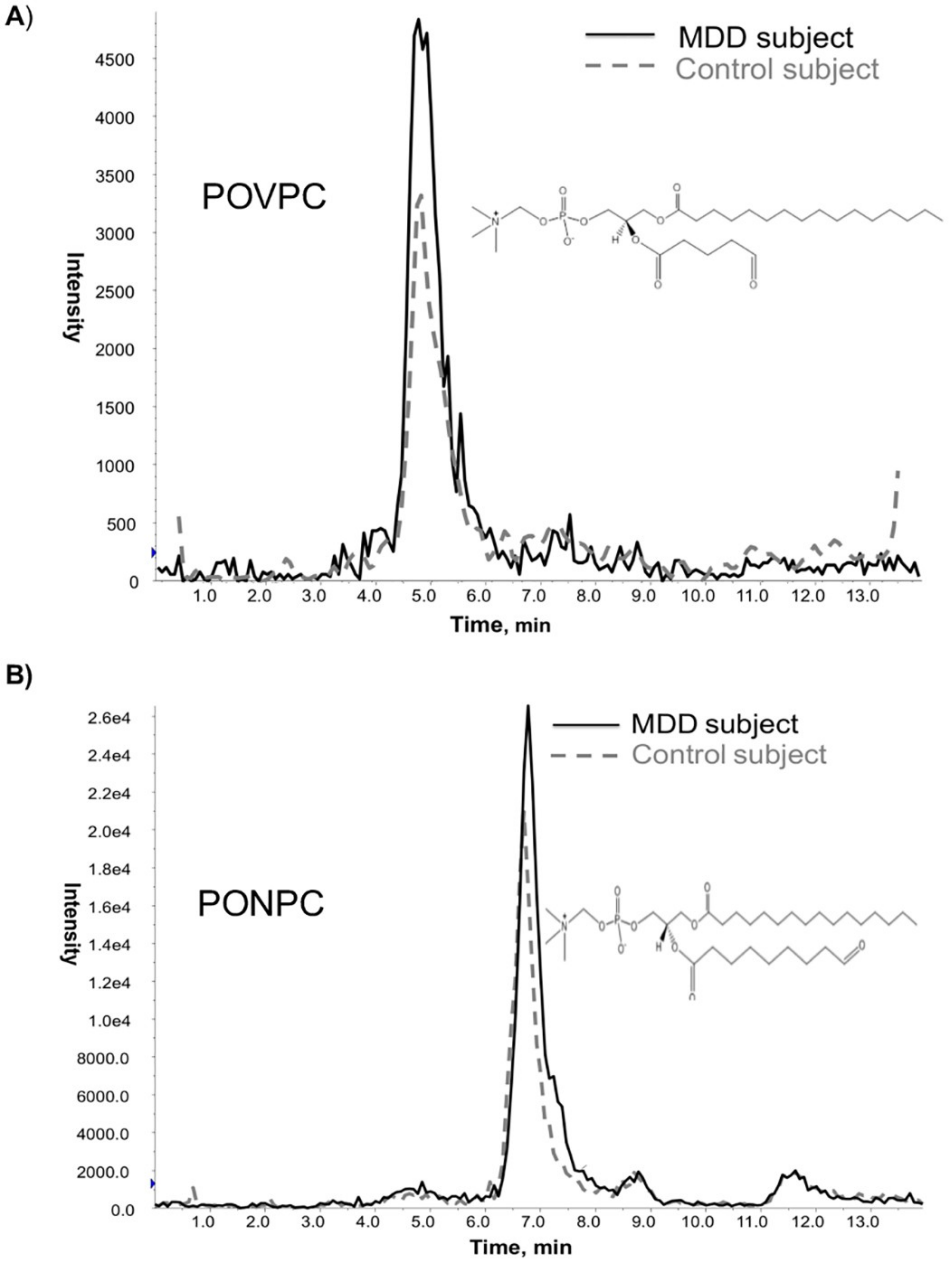
Multiple Reaction Monitoring [MRM] chromatogram of 2 fragmented OxPCs, [A] POVPC and [B] PONPC, in an MDD [black line] and control [dotted line] subject as measured by reverse phase HPLC-MS/MS. Abbreviations: POVPC, 1-palmitoyl-2-[5’-oxo-valeroyl]-sn-glycero-3-phosphocholine; PONPC,1-palmitoyl-2-[9’-oxo-nonanoyl]-sn-glycero-3-phosphocholine.

The separation and identification of OxPCs and oxylipins was carried out by reversed-phase high performance liquid chromatography [HPLC]. For OxPC analysis, samples were placed into a 4°C sample tray and subsequently injected onto an Ascentis Express C18 HPLC column [15 cm × 2.1 mm, 2.7 μm; Supelco Analytical, Bellefonte, Pennsylvania, USA] at 25°C using a Prominence HPLC system [Shimadzu Corporation, Canby, Oregon, USA]. The HPLC system was coupled to a 4000 QTRAP^®^ triple quadrupole mass spectrometer system with a Turbo V electrospray ion source from AB Sciex [Framingham, Massachusetts, USA]. For oxylipin analysis, evaporated samples were analysed by HPLC-MS/MS [API 4000, AB Sciex, Canada] as described in Monirujjaman et al. [28] Chromatographic and mass spectral data was collected using Analyst^®^ Software 1.6 [AB Sciex]. MultiQuant^®^ Software 2.1 [AB Sciex] was used to analyze the data. Ionization intensities of both OxPC and oxylipin species were based on the presence of the internal standard added prior to extraction. Relative amounts of the oxylipin and OxPC species were calculated by comparing the ratio of the peak area of the lipid to its internal standard peak area and multiplying it by the amount of internal standard present. Fig 2 represents the HPLC chromatograms displaying the relative peak intensities of two prevalent OxPC compounds in the MDD and control groups. Only signals having ionization intensities greater than five times the baseline noise were used for quantitation. Final results are presented as amount of OxPC or oxylipin in nanograms per μL of plasma.

### Statistical analysis

All statistical analyses were performed using SPSS Software version 24 [IBM Corporation, Armonk, NY, USA]. Significant changes were denoted when P<0.05. For demographics data, age differences between remitters, non-remitters and controls was analyzed using an analysis of variance [ANOVA] with Tukey post-hoc tests, while gender was assessed with a Fisher Exact Probability Test with Freeman Halton extension. Co-morbidities and treatment type differences between remitters and non-remitters was assessed with Chi-Square analysis, while pre and post Ham-D scores were compared with an Independent Samples T-test. An Independent Samples T-test was used to compare OxPCs in MDD subjects and controls, as well as oxylipins between remitters and non-remitters. Statistical analysis for baseline OxPC differences between remitters, non-remitters and controls was determined using a one-way ANOVA followed by Tukey post hoc tests. A Paired Samples T-test was used to compare pre and post-treatment OxPCs in all MDD subjects, as well as separately in remitter and non-remitter groups. Only 38 MDD subjects received a follow up blood draw post-rTMS treatment, thus subjects without post-rTMS values were excluded entirely from the pre/post analysis.

## Results

### Demographics

Demographics and clinical information is summarized in Table 1. Post-rTMS treatment, 22 patients were classified as remitters and 26 as non-remitters. Remitters and non-remitters were well matched in terms of gender and age. While no statistically significant differences were found between any groups in terms of gender [*p* = 0.237], it should be noted that the control group did have eight females and one male. Further, age was significantly lower in control subjects compared to both remitters [*p* = 0.046] and non-remitters [*p* = 0.009]. Remitters tended to have lower pre-treatment [*p* = 0.035] and post-treatment [*p* = 0.000] Ham-D scores. Remitters and non-remitters did not differ significantly in terms of co-morbidities or by treatment type provided. Co-morbidities in the nine control subjects included one patient with diabetes and asthma, and another with minor pulmonary hypertension.

**Table 1:** Demographics and clinical information of all subjects separated by remitter/non-remitter status and controls.

	Controls	Remitter	Non-remitter
N	9	22	26
Age [years, mean±SD]	33.1 ± 10.5*	46.6 ± 13.7	50.5 ± 16.6
Gender [% Female]	88.9%	54.5%	57.7%
Co-morbidities [%]			
Seizures		4.54%	0.0%
Hypertension		27.3%	30.1%
Heart attack		4.54%	3.8%
Stroke		0.0%	7.7%
Diabetes		4.54%	7.7%
Concussion		22.7%	23.1%
Treatment type [%]			
High		27.2%	23.1%
Theta		63.6%	73.1%
Low		9.09%	3.8%%
Pre-treatment Ham-D [mean±SD]		16.3 ± 3.7*	18.7 ± 4.1
Post-treatment Ham-D [mean±SD]		4.0 ± 2.3*	14.0 ± 5.5

*p<0.05.

### Baseline OxPC comparisons: MDD subjects vs. controls

**Fig 3:**
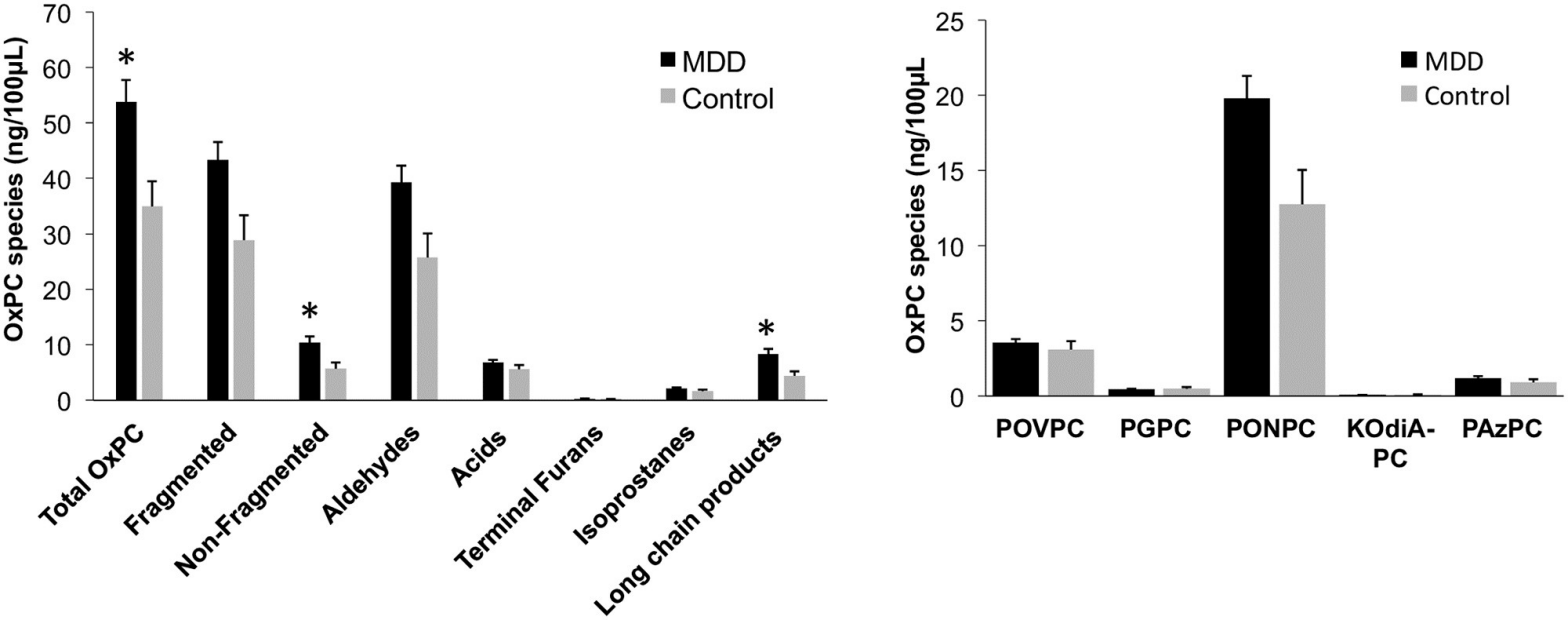
Baseline OxPC levels divided into total OxPCs with subgroups [A] and 5 specific OxPC compounds [B] compared between MDD subjects [n = 38] and controls [n = 9]. Values are mean±SEM. * = p<0.05.

**Fig 4:**
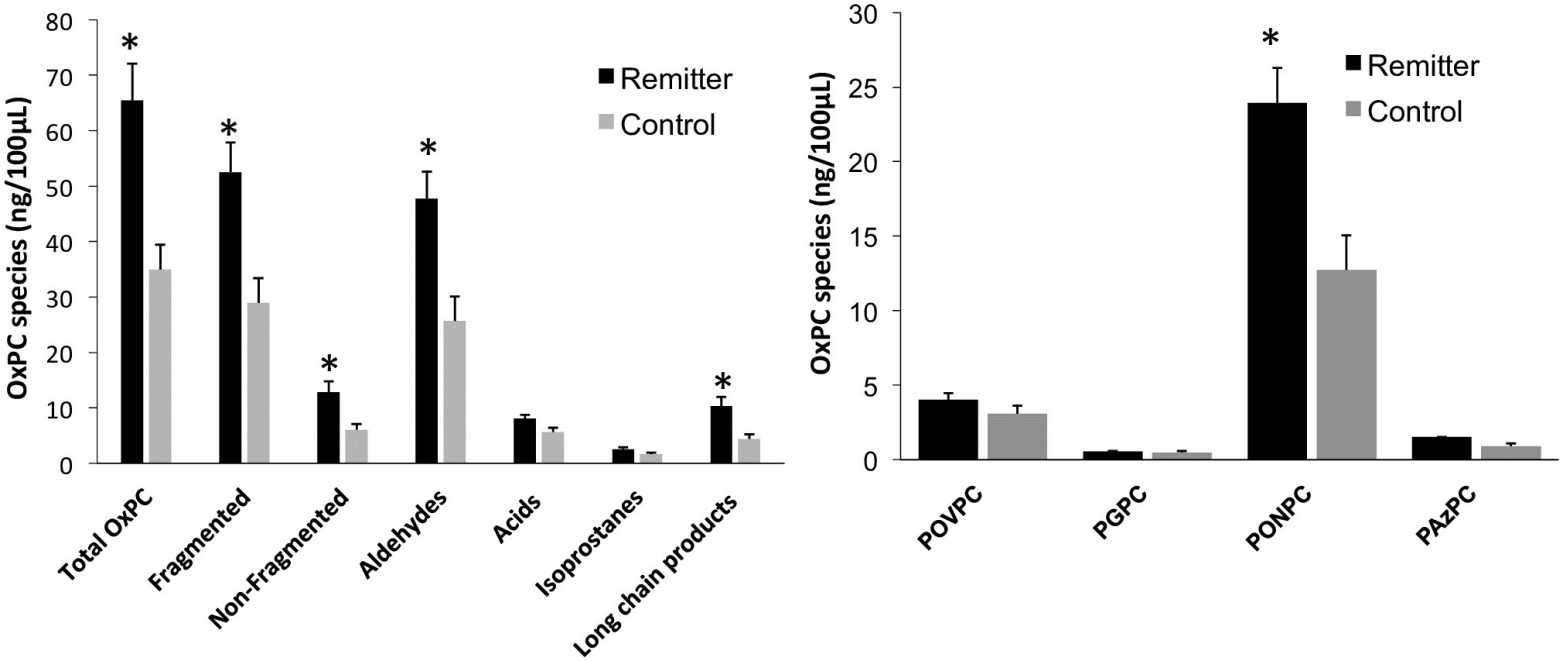
Baseline OxPC levels divided into total OxPCs with subgroups [A] and 5 specific OxPC compounds [B] compared between MDD subjects who entered remission after rTMS [n = 22] and control subjects [n = 9]. Values are mean+SEM. * = p<0.05.

Mass spectral analysis identified 57 distinct OxPCs in human plasma. These included a variety of fragmented OxPCs [aldehydes and carboxylic acids] and non-fragmented OxPCs [terminal furans, isoprostanes and long chain products]. OxPC species were compared between all MDD subjects and controls at baseline [Fig 3]. Non-fragmented OxPCs and long-chain products were found to be significantly higher in MDD subjects compared to controls [*p* = 0.006 and *p* = 0.003, respectively]. When MDD subjects were further separated into their respective treatment response groups, several OxPCs were found to be lower in control subjects compared to remitters, including total OxPCs [*p* = 0.007], fragmented [*p* = 0.011], non-fragmented [*p* = 0.044], aldehydes [*p* = 0.011], long-chain products [*p* = 0.029] and PONPC [*p* = 0.010] [Fig 4]. No significant differences were found between control subjects and non-remitters.

### Baseline OxPC comparisons: Remitters vs. non-remitters

**Fig 5:**
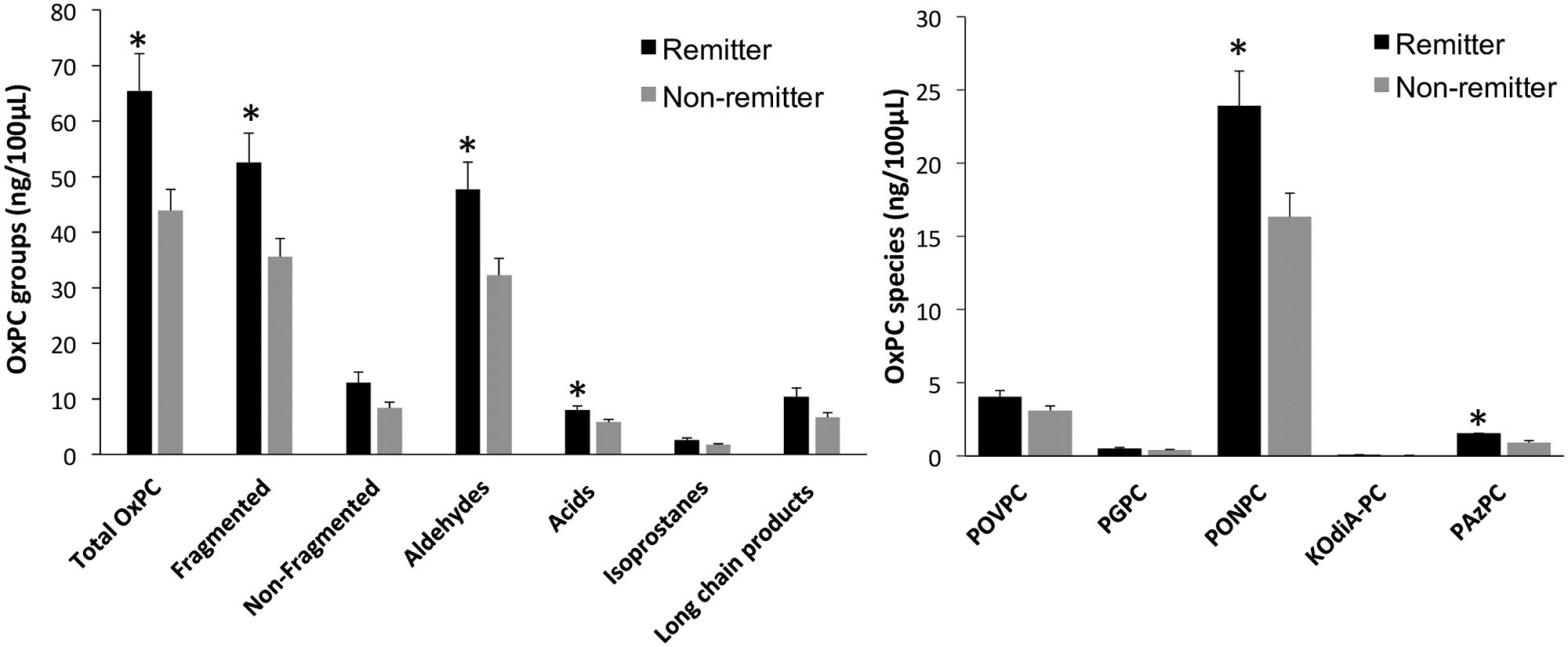
Baseline OxPC levels divided into total OxPCs with subgroups [A] and 5 specific OxPC compounds [B] compared between MDD remitters [n = 22] and non-remitters [n = 26]. Values are mean+SEM. * = p<0.05.

Pre-rTMS OxPC levels were compared between remitters and non-remitters [Fig 5]. Total OxPC was found to be significantly higher in remitters compared to non-remitters at baseline [*p* = 0.009]. When further divided into OxPC sub-groups, remitters had significantly elevated fragmented OxPCs [*p* = 0.013], aldehydes [*p* = 0.015] and carboxylic-acids [*p* = 0.028]. The two specific OxPCs that were significantly higher in remitters compared to non-remitters were PONPC [*p* = 0.019] and 1-palmitoyl-2-azelaoyl-sn-glycero-3-phosphocholine [PAzPC] [*p* = 0.040].

### Pre- to post-OxPC changes based on treatment response

**Fig 6:**
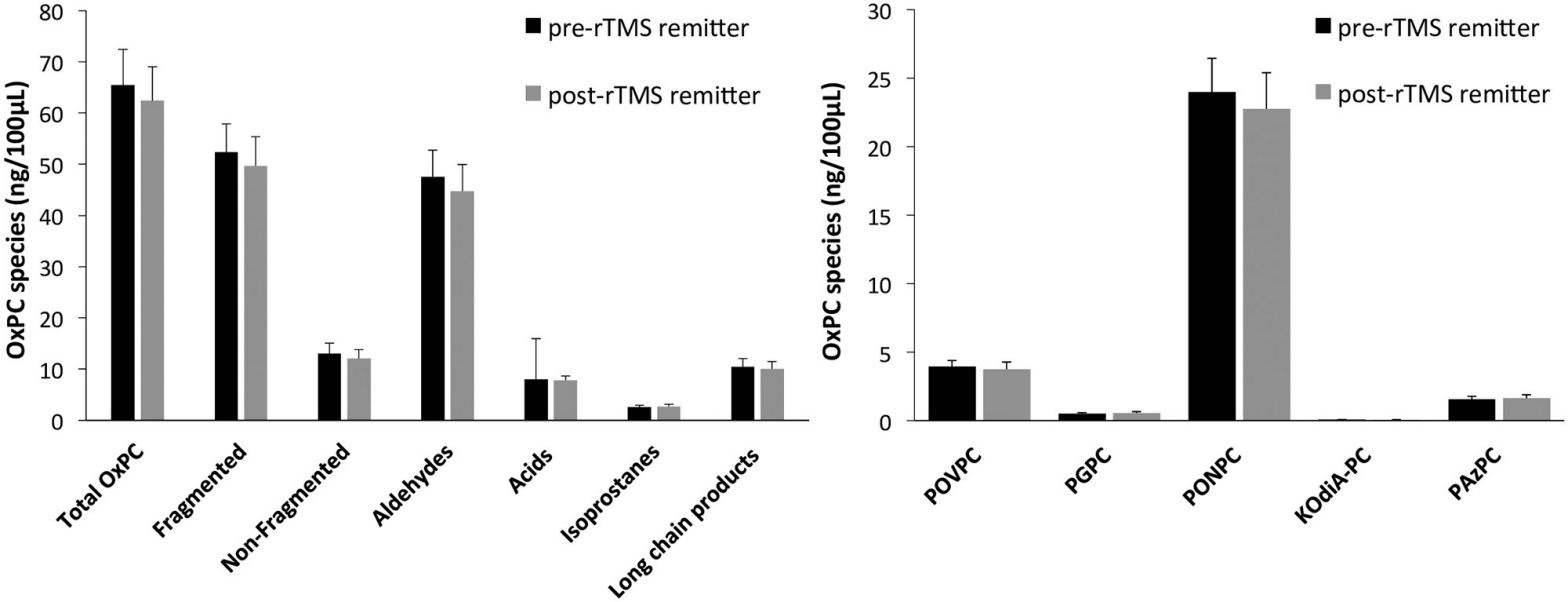
OxPC levels divided into total OxPCs with subgroups [A] and 5 specific OxPC compounds [B] compared between MDD remitters pre-rTMS [n = 21] and post-rTMS [n = 21]. Values are mean+SEM.

**Fig 7:**
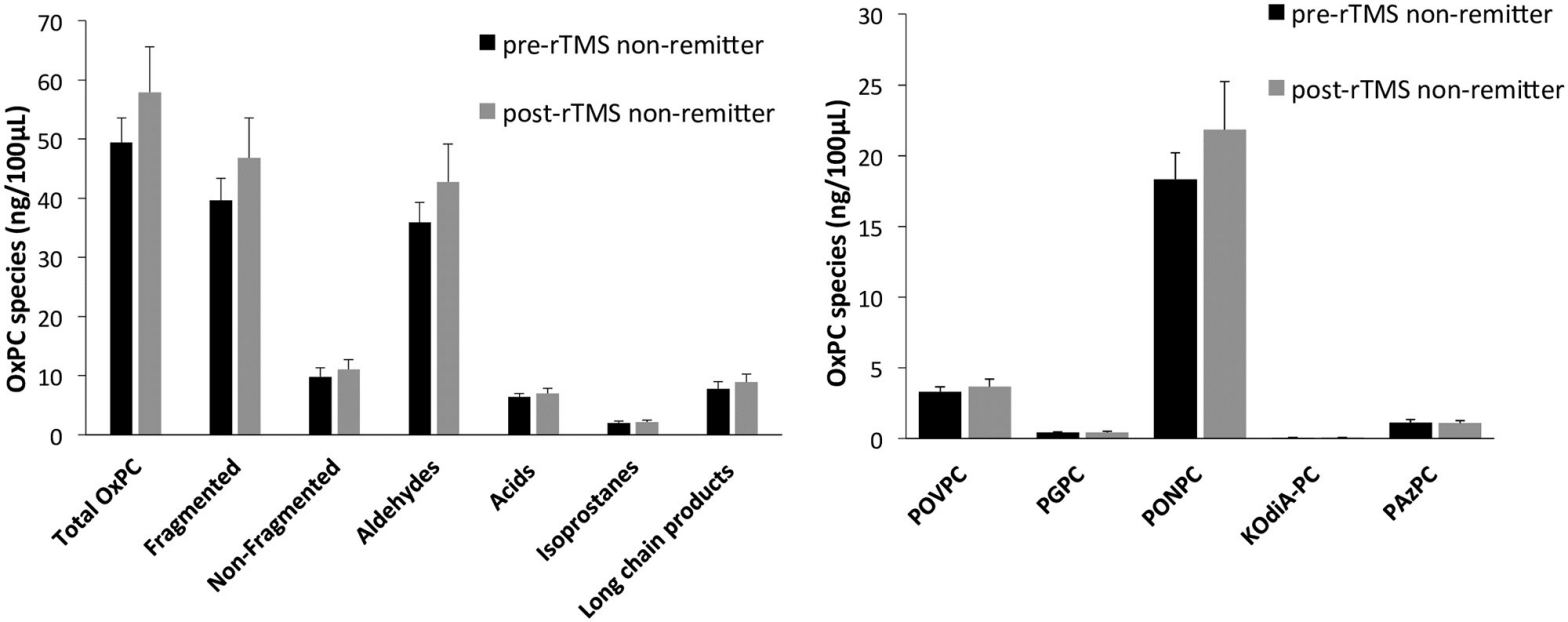
OxPC levels divided into total OxPCs with subgroups [A] and 5 specific OxPC compounds [B] compared between MDD non-remitters pre-rTMS [n = 17] and post-rTMS [n = 17]. Values are mean+SEM.

MDD subjects showed no significant changes in OxPC levels after treatment with rTMS. When MDD subjects were separated into their treatment response groups, there were no significant changes in OxPC levels after rTMS treatment in remitters [Fig 6], nor in non-remitters [Fig 7].

### Baseline oxylipin comparisons: Remitters vs. non-remitters

Mass spectral analysis identified 32 distinct oxylipin species in human plasma of remitters and non-remitters before rTMS treatment (Table 2). When comparing pre-rTMS oxylipin values, no significant differences were found between remitters and non-remitters in any of the compounds identified. Based on the lack of differences at baseline, post-rTMS values were not analyzed.

**Table 2:** Mean values [±SEM] of all oxylipin species [ng/ul] identified in remitters [n = 21] and non-remitters [n = 25] before rTMS treatment.

Oxylipin species	Pathway	FA	Remitter	Non-remitter
11,12 DiHETrE	CYP	AA	0.21 ± 0.02	0.24 ± 0.02
14,15 DiHETrE	CYP	AA	0.26 ± 0.02	0.30 ± 0.02
19,20 DiHDoPE	CYP	DHA	0.11 ± 0.01	0.13 ± 0.01
5,6 DiHETrE	CYP	AA	0.02 ± 0.00	0.02 ± 0.01
12,13 diHODE	CYP	LA	1.18 ± 0.17	1.19 ± 0.19
12,13 diHOME	CYP	LA	22.40 ± 4.82	21.84 ± 5.00
12,13 EpODE	CYP	LA	0.21 ± 0.03	0.22 ± 0.04
12,13 EpOME	CYP	LA	3.82 ± 0.71	4.47 ± 1.27
15,16 EpODE	CYP	LA	1.01 ± 0.20	1.17 ± 0.30
9,10 diHOME	CYP	LA	26.86 ± 4.2	20.54 ± 2.9
9,10 EpOME	CYP	LA	1.19 ± 0.19	1.49 ± 0.36
9,10,13 triHOME	LOX/CYP	LA	19.11 ± 2.93	16.28 ± 2.60
9,12,13 triHOME	LOX/CYP	LA	25.15 ± 3.21	20.88 ± 3.51
13-HODE	LOX	LA	8.74 ± 1.81	7.58 ± 1.39
13-HOTrE	LOX	ALA	0.54 ± 0.08	0.69 ± 0.15
dihomo PGJ2	COX	ADA	0.81 ± 0.18	1.65 ± 1.07
9-HODE	LOX	LA	3.88 ± 0.74	3.26 ± 0.57
9-HOTrE	LOX	ALA	0.71 ± 0.09	0.93 ± 0.20
20cooh AA	CYP	DHA	1.97 ± 0.33	1.98 ± 0.24
20-HDoHE	CYP/NE	LA	0.03 ± 0.00	0.04 ± 0.00
13-oxoODE	LOX	AA	0.19 ± 0.05	0.15 ± 0.02
15-oxoETE	LOX	LA	0.02 ± 0.00	0.03 ± 0.01
9-oxoODE	LOX	AA	0.34 ± 0.07	0.28 ± 0.05
11-HETE	NE/COX/LOX	AA	0.15 ± 0.03	0.17 ± 0.02
Tetranor 12-HETE	LOX	AA	0.19 ± 0.02	0.16 ± 0.03
15-HETE	LOX	DGLA	0.09 ± 0.04	0.07 ± 0.01
15-HETrE	LOX	DHA	0.26 ± 0.22	0.07 ± 0.01
16-HDoHE	LOX	AA	0.08 ± 0.02	0.07 ± 0.01
16-HETE	CYP	DHA	0.06 ± 0.04	0.03 ± 0.01
4-HDoHE	LOX	AA	0.08 ± 0.01	0.10 ± 0.02
5-HETE	LOX	AA	0.15 ± 0.03	0.13 ± 0.02
8-HETE	LOX	DHA	0.09 ± 0.02	0.09 ± 0.01

Abbreviations: FA = Fatty acid precursor, LOX = lipoxygenase, CYP = cytochrome p450, NE = non-enzymatically, ALA = alpha-linoleic acid, LA = linoleic acid, DHA = docosahexaenoic acid, AA = arachidonic acid, DGLA = dihomo-γ-linolenic acid, ADA = adrenic acid.

## Discussion

This is the first study to compare enzymatic and non-enzymatic oxolipidomics profile between non-depressed control subjects and patients with MDD before and after rTMS treatment. Our study revealed that MDD subjects had higher pre-rTMS levels of certain OxPC subgroups when compared to non-depressed controls. As OxPCs are known to be both biomarkers and mediators of oxidative stress, our results support the growing body of evidence that oxidative stress, and associated inflammation, may be a contributing factor to the pathophysiology of MDD [10, 29]. For example, fragmented OxPCs are biologically active molecules that can be recognized by the innate immune system via toll-like receptors, scavenger receptors and natural antibodies [14, 30]. Further, OxPCs can cause a variety of harmful effects on the brain and body, including neuroinflammation, apoptosis and altered neurotransmitter metabolism [31]. A recent meta-analysis highlighted how individuals with MDD show elevated levels of oxidative stress and decreased anti-oxidants in the brain post-mortem [4]. Moreover, oxidative stress is known to have inhibitory effects on neurogenesis, and as clinical depression is associated with decreased gray-matter volume in multiple brain regions, this provides a biological mechanism for how inflammation and oxidative stress may contribute to clinical depression [32, 33]. Additionally, chronic low-grade inflammation and pro-inflammatory cytokines can affect many physiological domains relevant to depression, such as the induction of sickness behavior and fatigue [34]. Further, OxPCs play a crucial role in the development of atherosclerosis, and since depression and cardiovascular disease are highly co-morbid diseases, this suggests the two may share common underlying pathologies [3, 16].

Importantly, the present study’s finding that when separated into their respective treatment response groups, *only* MDD remitters, and not non-remitters, differed from controls, supports the theory that only certain cases of MDD may have underlying oxidative stress and inflammation. Indeed, the literature supports that the association between inflammation, oxidative stress and depression is not consistent among all forms of MDD. Hickman et al. found that the inflammatory marker CRP was elevated only in patients with atypical depression. Similarly, atypical depression showed elevated markers of inflammation and metabolic dysregulation compared to melancholic depression [34]. Moreover, only in certain subgroups of MDD, particularly ones with elevated peripheral inflammatory markers, are depressive symptoms alleviated with treatments such as non-steroidal anti-inflammatories [NSAIDS] and anti-TNF medications [35]. While lifestyle factors such as smoking, alcohol use and BMI likely contribute to differences in inflammation and oxidative stress among depressed patients, a meta-analysis done on the relationship between oxidative stress and depression maintains that there is an association that persists independent of these lifestyle variables [4].

The primary objective of this study was to determine if there were differences in oxolipidomics profile between people who entered remission after rTMS and those who did not. Our exploratory analyses showed that remitters had significantly higher levels of OxPCs than non-remitters before treatment. Overall, it appears that patients with MDD who have higher levels of oxidative stress, as revealed by OxPC biomarker analysis, tend to enter remission after rTMS more so than individuals with lower levels of oxidative stress. While a recent review did highlight various other factors that have been associated with a positive response to rTMS, including higher metabolic activity of the left DLPFC, improved connectivity between the DLPFC and striatum, high anterior cingulate cortex volume, and lower baseline glutamate levels, to our knowledge, no other studies have investigated oxidative stress as a predictor of rTMS response [36].

Additionally, our OxPC results contribute to the growing body of knowledge in how rTMS may be working to alleviate depressive symptoms. Studies using animal models have shown that rTMS has a variety of effects on the brain, including increases in NMDA-receptor density, brain-derived neurotrophic factor [BDNF], regional cerebral blood flow, dopamine, serotonin, activation and migration of astrocytes, as well as decreases in brain cortisol levels and apoptotic markers [37, 38]. However, many studies have noted that rTMS also works to reduce oxidative stress and inflammation in the brain [19–22]. In rats with oxidatively stressed brains, rTMS increased the antioxidants glutathione [GSH], GSH-peroxidase and catalase, as well as decreased lipid peroxidation products [19]. In patients with Parkinson’s Disease, rTMS reduced plasma pro-inflammatory cytokines INF-y and IL-17a [20]. Importantly for the present study, preliminary *in vitro* studies have shown that magnetic stimulation can have a protective effect against oxidative stress, suggesting that oxidative stress may be targeted by rTMS as part of its therapeutic effect [19]. If individuals who enter remission have higher baseline oxidative stress, perhaps their depression is, in part, attributable to elevated levels of oxidative stress, and rTMS is functioning to reduce it. In other words, rTMS may work better in individuals with high levels of oxidative stress and inflammation since it has this added biological mechanism to function through.

In fact, several studies have hypothesized that inflammation may be one reason some individuals don’t respond to conventional antidepressant treatments. For example, Lindqvist et al. looked at F2-isoprostanes and 8-OHdG as predictors of treatment response to SSRIs [29]. The study found that F2-isoprostanes were higher in non-responders compared to responders. These results are opposite to the findings of the present study, where oxidative stress was higher in treatment responders. However, this is actually concordant with the present study’s results, reinforcing the established evidence that rTMS may work to treat depression through different mechanisms than classic pharmacotherapies like SSRIs [38]. This may explain why individuals with treatment resistant depression can be helped by rTMS even after failing multiple courses of antidepressants. Indeed, much research has linked inflammation, and by extension oxidative stress, to antidepressant treatment non-responsiveness [39].

Interestingly, no differences in any oxylipins were found between remitters and non-remitters. Thus, it appears the biochemical variations found to be different among these two groups is primarily due to non-enzymatic processes, as oxylipins [unlike OxPCs] are produced enzymatically. Another potential reason for the lack of oxylipin differences is that patients were not asked to fast prior to blood draws or report recent medication consumption such as non-steroidal anti-inflammatories [NSAIDS]. Both of these factors can cause alterations in oxylipin metabolism and profiles.

Irrespective of clinical response, no changes in OxPC levels were detected as a result of rTMS treatment. There are several possible reasons for the lack of change in OxPCs post-rTMS. OxPCs only represent one set of biomarkers for oxidative stress [14]. Thus, it is possible rTMS may have diffuse and widespread effects on the brain that are too difficult to detect by solely examining OxPCs. A second reason may be that brain changes in oxidative stress lipid profiles are not transmitted to the plasma immediately after finishing treatment. Blood draws were performed either immediately or within 24 hours of finishing rTMS treatment; thus, it is possible that changes occurring within the brain were not detectable yet as free OxPCs in plasma.

There are several limitations to the present study. First is the small sample size of control subjects compared to MDD subjects. Due to this small sample size, OxPC values of healthy controls may not be completely representative of the larger population. Further, control subjects were significantly younger than MDD patients. As oxidative stress increases with age, this is a potential confounding factor to our results [40]. Secondly, metabolic data including BMI, smoking status and alcohol use was not recorded for any subjects. Oxidative stress and inflammation are intimately related with these factors and would ideally be controlled for in statistical analyses. Having said that, no significant differences were found in the co-morbidities that were reported between remitters and non-remitters. Thirdly, many non-remitter subjects refused follow up blood draws after rTMS treatment. This resulted in a slightly smaller sample size when pre- and post-treatment analyses were conducted, potentially resulting in non-statistically significant results. Lastly, almost all MDD subjects were on a variety of psychotropic medications during rTMS, which may have effects on oxidative stress biomarkers.

## Conclusions and future directions

The heterogeneity in treatment response and biochemical variations in patients with MDD, as well as the association between rTMS and the reduction of oxidative stress, was the reason for investigating patient oxolipidomics profile in prediction of treatment response to rTMS. Our findings are consistent with the growing body of research that certain subgroups of MDD exhibit elevated levels of oxidative stress and inflammation [35, 41]. Additionally, MDD patients with higher levels of oxidative stress, as revealed by OxPC biomarker analysis, appear to respond better to rTMS than those with lower levels of oxidative stress. Perhaps, patients with this phenotype of depression may be more receptive to rTMS treatment. In the future, with larger scale studies and predictive-type analyses, OxPC and lipidomics analysis may be able to predict who will respond to rTMS through a simple blood test. Not only would this would drastically decrease wait times for rTMS, but also would reduce healthcare costs wasted on ineffective treatment methods. Additionally, larger scale studies with stricter fasting criteria should be conducted to examine differences in oxylipin profiles between MDD patients, both pre and post-rTMS, and control subjects. Conducting more research of this nature, geared towards precision and personalized medicine, will only further improve the lives of patients and the healthcare system at large.

## Supporting information

S1 Data Mass spectrometric data. (XLSX)

## Abbreviations

AA: arachidonic acid
ADA: adrenic acid
ALA: alpha-linoleic acid
COX: cyclooxygenase
CYP: cytochrome p450
DGLA: dihomo-γ-linolenic acid
DHA: docosahexaenoic acid
DLPFC: dorsolateral pre-frontal cortex
ECT: electroconvulsive therapy
FA: Fatty acid
KOdiA-PC: 1-palmitoyl-2-[5’-keto-6’-octenedioyl]-sn-glycero-3-phosphocholine
LA: linoleic acid
LC-MS: liquid chromatography mass spectrometry
LOX: lipoxygenase
MDD: Major Depressive Disorder
NE: non-enzymatically
OxPL: oxidized phospholipids
PAzPC: 1-palmityl-2-azelayl-sn-glycero-3-phosphocholine
PGPC: 1-palmitoyl-2-glutaryl-sn-glycero-3-phosphocholine
PONPC: 1-palmitoyl-2-[9’-oxononanoyl]-sn-glycero-3-phosphocholine
POVPC: 1-palmitoyl-2-[5’-oxo-valeroyl]-sn-glycero-3-phosphocholine
PUFAs: polyunsaturated fatty acids
ROS: reactive oxygen species
rTMS: repetitive transcranial magnetic stimulation
TRD: treatment resistant depression
